# Youth awareness on death and dying in school settings: A scoping review on knowledge and practices

**DOI:** 10.1177/26323524261461310

**Published:** 2026-06-16

**Authors:** Emilie Allard, Clémence Coupat, Dimitri Létourneau, Gabrielle Fortin, Olivia Nguyen, Sabrina Lessard

**Affiliations:** 1 Faculty of Nursing, 5622 Université de Montréal, Montréal, QC, Canada; 2 Research center, Centre intégré universitaire de santé et de services sociaux (CIUSSS) du Nord-de-l’Île-de-Montréal, Montréal, QC, Canada; 3 School of Social Work and Criminology, Laval University, Québec, QC, Canada; 4 Research center, 4440 CHU de Québec-Université Laval, Québec, QC, Canada; 5 Department of Family and Emergency Medicine, 5622 Université de Montréal, Montréal, QC, Canada; 6 Palliative care division, CIUSSS du Nord-de-l’Île-de-Montréal, Montréal, QC, Canada; 7 Centre for Research and Expertise in Social Gerontology, CIUSSS Centre-Ouest-de-l'Île-de-Montréal, Montréal, QC, Canada; 8 Department of Anthropology, 5622 Université de Montréal, Montréal, QC, Canada

**Keywords:** death education, death and dying, school curriculum, death literacy, children, adolescent, community engagement, end-of-life discussion, palliative care

## Abstract

**Background:**

School is a key setting for a child’s cognitive, social, and civic development and may therefore offer a promising space to open dialogue about death and dying. Yet awareness-raising practices on death and dying in schools can be difficult to identify within the abundance of scientific and practical writings. To support school staff and enrich curricula with coherent and context-sensitive approaches, we sought to map existing practices to better understand the levers, barriers, and conditions for success.

**Objectives:**

To explore the current state of knowledge and practices on awareness about death and dying (ADD) in the school context, and answer these questions: 1) How is ADD addressed in school settings? 2) What are the views of young people, parents, and school personnel on raising ADD? 3) What factors influence such ADD?

**Design:**

This scoping review followed Levac’s methodology.

**Methods:**

Searches were performed in 17 scientific and grey literature databases, complemented by reference tracking and community partners’ consultation. Data were charted by research questions and analyzed using content analysis.

**Results:**

35 writings (2010-2023) were included. Various awareness-raising practices combining multiple activities were identified, emphasizing the value of multimodal approaches that engage cognitive, emotional, and creative dimensions. Interdisciplinary collaboration with community organizations was frequently requested to support ADD practices. Perceptions of ADD were generally positive, though nuances emerged among young people, school personnel and parents. Our results identify numerous factors that influence ADD, span between individual, family, school, and sociocultural levels, showing the multidimensionality and systemic nature of this phenomenon.

**Conclusion:**

ADD in schools is shaped by its context, such as collaboration between interested parties, teacher training, sensitive practices, and supportive policy frameworks enabling meaningful curricula integration. Without clear political and institutional support, initiatives remain fragile and dependent on isolated actors.

## Plain language summary

Death is part of life, yet many children say it is rarely spoken about at school. We reviewed recent publications to better understand how awareness about death and dying (ADD) in school settings was viewed and practised. We found 35 sources published between 2010 and 2023.

**1) How do we raise ADD?** A range of activities can be used in school settings: guided class discussions, short talks by nurses, psychologists, or community workers, creative projects like drawing or storytelling, films or books to start a conversation, and, in some cases, visits to hospices or community spaces. In the reviewed writings, combining activities was found to help children think, feel, and express themselves.

**2) What are the views of children, parents, and school staff on raising ADD?** In the included writings, children often described death as being *everywhere* but not openly discussed. In most of the writings that reported on parents’ and school staff’s perspectives, they were supportive, even if they expressed some nuances and concerns.

**3) What factors influence ADD?** The writings reviewed suggest that ADD is influenced by the child’s age, cognitive development and grief experiences, family’s willingness to talk about death, school environment, curriculum, as well as teachers’ confidence, and more broadly culture, religion, media, and local policies. Because of these different factors, the integration of ADD in school requires clear guidance and teacher training, and cooperation with communities can make a real difference.

In short, the writings suggest that school settings can provide safe opportunities for children to find words, ask questions, and build “death literacy”. When thoughtfully integrated, such practices may help young people face end-of-life realities with greater understanding and for themselves and others.

## Background

The report of the *Lancet Commission on the Value of Death*^1^ highlights the ambivalent relationship to death and dying that contemporary societies maintain, particularly in high-income countries. While death denotes the precise moment when life ends, defined as the total and irreversible cessation of cardiorespiratory and brain functions,^2^ dying is a process embedded within individual and collective trajectories and is situated within a broader and evolving societal context. Considered a social construct, this process offers an outstanding lens through which we can better understand social dynamics, as dying is imbued with cultural and political meanings that vary by time and place.^3,4^

Addressing death and dying remains difficult, as these phenomena are so emotionally charged that they are often described as taboo.^1,5^ The topic is even more challenging to address with children. Adults’ responses must consider factors such as age, developmental stage, personality, and religious beliefs, which may shape young people’s understanding of death and dying.^6–8^ Young people do not encounter death and dying in a vacuum; they make sense of these experiences within the social and cultural worlds they inhabit,^9^ drawing on their own bereavement experiences (e.g., the death of a relative or a pet) and on the constant presence of death-related images and narratives in contemporary media.^6,7,10–13^ This hesitancy is also reflected in the first author’s parental experience, when she was invited to give a talk about palliative care at her daughters’ elementary school. This experience sparked the present research project, as the children emphasized that they valued open discussions about death and dying, but felt that not enough space was dedicated to the subject in the school curriculum.

Schools are a key setting for fostering young people’s cognitive, emotional, social, and civic development. By bringing together children and adolescents from diverse cultural, religious, and social backgrounds, schools provide a unique space to open dialogue on sensitive and universal topics such as death and dying.^14^ It is with this view of fostering dialogue that the field of “death education” has emerged, referring to educational practices aimed at developing individuals’ knowledge, reflexivity, and capacity to act in relation to dying, death, and grief.^15^ However, few studies have explicitly addressed death education for children in a school context, prior to a critical situation involving the death of a member of the school community. In this review, we focus on proactive and anticipatory forms of death education rather than on crisis-oriented responses to an actual death in the school community. For this reason, we adopt the broader term *awareness about death and dying* (ADD), which we define as any practice that seeks to raise young people’s awareness, foster their reflexivity, open discussion, and provide a safe environment for them to ask questions about death, dying, or grief. This working definition, developed by our team for the purposes of this review, makes it possible to include not only initiatives formally identified as death education, but also other pedagogical and relational experiences that contribute to strengthening death literacy.^16^ In line with Noonan et al.,^16^ death literacy refers to “a set of knowledge and skills that make it possible to gain access to, understand, and act upon end-of-life and death care options” (p.32); it is a resource that both individuals and communities can draw upon. We hypothesized that school-based ADD practices may contribute to developing early forms of death literacy among young people.

Indeed, Sallnow et al.^1^ emphasize the urgency of transforming social representations of death and dying, and of encouraging educational initiatives that foster a better understanding of these universal realities. While various, scattered school initiatives^17^ do exist, there appears to be no overarching vision or consolidated resources to guide school staff in proactively addressing death and dying with students. This lack of reference points reinforces the idea that death is too delicate a topic for schools, even though young people are confronted by it daily, through personal experiences or the media.

To develop practices that are coherent, context-sensitive, and evidence-based, that can support school staff and enrich curricula, we first need to better understand and map existing awareness practices to identify the levers, barriers, and conditions for success.

## Objectives

The objective of this study is to explore the current state of knowledge and practices on ADD in the school context. More specifically, it seeks to address three questions:

1. How is awareness about death and dying addressed in school settings?
2. What are the views of young people, parents, and school personnel on raising awareness about death and dying in school settings?
3. What factors influence such awareness-raising in school settings?

## Methods

This article follows the structure (Supplementary file 1) proposed by the Preferred Reporting Items for Systematic Reviews and Meta-Analyses (PRISMA) extension for Scoping Reviews (PRISMA-ScR).^18^ The six stages of the Levac et al.^19^ framework were followed: 1) identify the review questions, 2) identify literature, 3) select literature, 4) extract data, 5) report results, and 6) consult interested parties. Since the protocol of this scoping review was published in an open-access, peer-reviewed journal,^20^ and the research questions have already been presented, the five remaining stages are described briefly. At least four members of our research team were involved at every stage of the review process, and methodological decisions were discussed within the broader research team.

## Identify literature

The search strategy combining keywords and thesaurus terms was developed in partnership with a health sciences librarian and is available in the published protocol.^20^ The following databases were searched: *CINAHL Complete* (EBSCO), *MEDLINE* (Ovid), *EBM Reviews Cochrane* (Ovid), *JBI EBP Database* (Ovid), *PsycINFO* (Ovid), *Web of Science* (Clarivate), *Global Health* (Ovid), *Sociological Abstracts* (ProQuest), *Social Sciences Abstracts* (EBSCO), *Family Studies Abstracts* (EBSCO), *Social Services Abstracts* (ProQuest), *Social Work Abstracts* (EBSCO), *Érudit*, *CAIRN*, and *PubPsy*. Grey literature was searched systematically through *Dissertations & Theses Global* (ProQuest) and *Google Scholar*. Table 1 provides an example of the search strategy in *CINAHL*. The database search was completed on June 25, 2023. Reference lists of included publications were manually screened, and collaborators were consulted to identify additional nonindexed materials.

**Table 1.** Equations with thesaurus and keywords in the CINAHL Complete (EBSCO) database.

​	Concept: Awareness/education about dying and death		Population: Young people		Context: School	
Equation with thesaurus	T1	((MH “Death Education”) OR (MH “Attitude to Death”) OR (MH “Death+”) OR (MH “Terminal Care+”) OR (MH “Bereavement+”) OR (MH “Terminally Ill Patients”) OR (MH “Hospice Patients”) OR (MH “Hospices”) OR (MH “Suicide, Assisted”) OR (MH “Burial Practices”))	T2	(MH “Child”) OR (MH “Adolescence”)	T3	(MH “Schools, Middle”) OR (MH “Schools, Elementary”) OR (MH “Schools, Secondary”) OR (MH “Schools, Special”) OR (MH “Teachers”) OR (MH “Curriculum+”) OR (MH “Students, High School”) OR (MH “Students, Middle School”) OR (MH “Students, Elementary”)
Equation with keywords - Fields: (TI, AB, MW)	K1	(( Death* OR Dying OR Palliati* OR Hospice* OR Euthanasia OR Bereav* OR bereft OR grief OR grieving OR mourning OR funeral* ) OR ( (Terminal* N1 (care OR ill*)) OR (suicide N2 assist*) ) OR ( “End-of life” OR “Supportive care” ))	K2	( Youth* OR Child* OR Boy* OR Girl* OR Kid OR Kids OR Adolescen* OR Teen* )	K3	(( School* OR Kindergarten* OR Curriculum* OR Teacher* OR Pupil*) OR ((Education OR Student*) N1 (Primary OR Secondary OR Elementary )))
Final equation	**((T1 OR K1) AND (T2 OR K2) AND (T3 OR K3))**					

***Notes.*** AB: abstract, MW: word in subject heading, TI: title. The asterisk (*) represents a truncation in CINAHL.

## Select literature

The selection process was conducted independently using *Covidence* (Veritas Health Innovation Ltd., Melbourne, Australia), a systematic review management platform. Before undertaking the two selection phases, team calibration exercises were carried out on 30 records (title and abstract) and 3 records (full text) to clarify inclusion criteria and selection tools. Discrepancies were discussed until consensus was reached. Inclusion and exclusion criteria are presented in Table 2 and detailed in the protocol.^20^

**Table 2.** Inclusion and exclusion criteria used for the selection of writings.

Eligibility criteria	Inclusion	Exclusion
Population	School-age children or teenagers Parents Teachers and school staff	Bereaved children or teenagers
Context	Elementary school (or equivalent Secondary school (or equivalent)	Kindergarten Informal education settings (i.e. daycare) Outside the institutional framework of a school (i.e. extracurricular or community activities)
Concept	Explicitly deals with awareness of death and dying in school settings	Grief support Suicide prevention Chronic illness as the central issue Bereavement not related to death (i.e. divorce)
Type of writings	Primary studies Literature reviews Grey literature Theoretical publications Practice stories	Blogs Media entries Personal opinions Book reviews Letters to the editor / Editorials Conference abstracts Research protocols
Language	French English	​
Publication date	01/01/2009 - 25/05/2023	​

**Figure 1.**
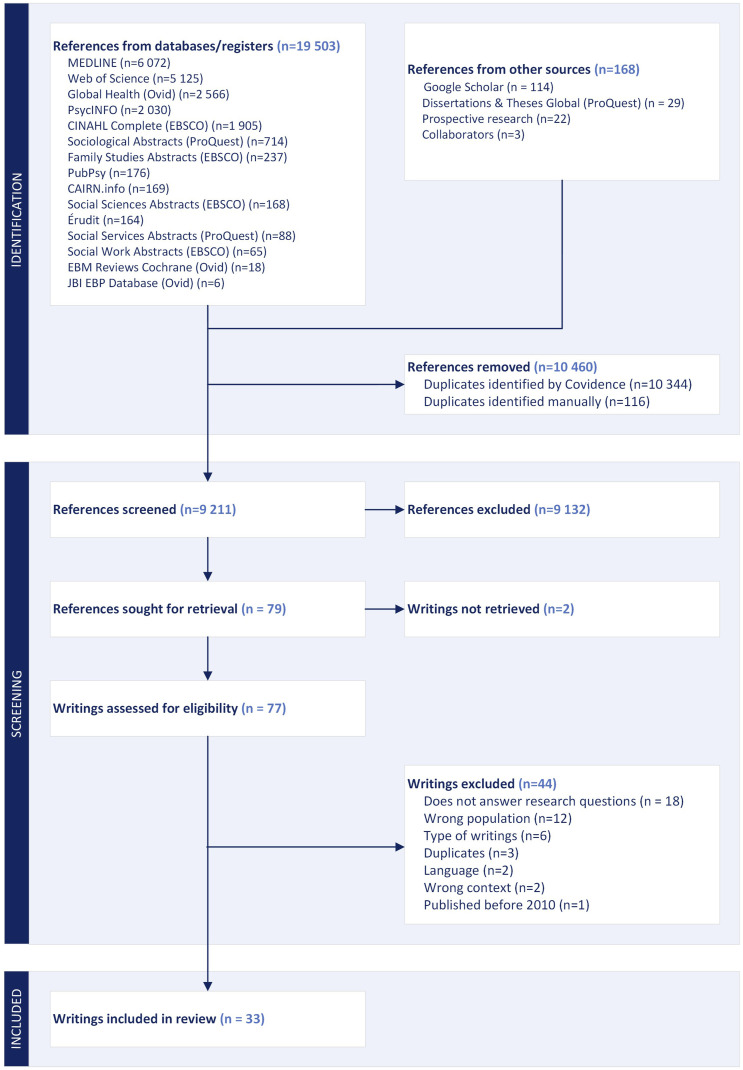
PRISMA data flowchart diagram.

The database search yielded 16,927 records. After the removal of duplicates, 9,220 records were screened against the eligibility criteria. Figure 1 presents the PRISMA flow diagram, detailing the number of publications selected at each stage and the main reasons for exclusion.

## Extract data and analyse

In *Covidence*, data extraction was performed by one co-author and validated by another, following a calibration exercise on three records to develop the extraction tool (Table 3). The methodological quality of the included publications was not assessed, given the diversity of document types included.^20^

**Table 3.** Data extracted for analysis.

General data	Theoretical data	Methodological data	Results		
			Interventions/practices	Percep-tions	Influencing factors
Title	Framework (if applicable)	Study design	Design	Young people	Facilitating factors
Year	​	Sample (size, inclusion and exclusion criteria, age, gender distribution)	Year	Parents	Constraining factors
Authors	​	School level	Duration	School staff	​
Discipline	​	Data collection method	Topics covered	​	​
Type of writings	​	Data analysis method	Practitioners involved	​	​
Aim/objectives	​	​	Barriers	​	​
Country	​	​	Facilitators	​	​
​	​	​	Suggestions for improvement	​	​

Raw data were organized according to their contributions to each research question. A content analysis^21^ was conducted in three steps: 1) coding, 2) identifying similarities and differences, and 3) identifying themes and subthemes. Codes were assigned to the extracted data according to the research question they answered. These codes were as close as possible to the words used by the authors. The codes were then compared to identify similarities and differences, both within and between population groups (young people, parents, or school staff). These comparisons identified unifying themes that guided the presentation of the results.

## Consult interested parties

The research team collaborated with community organizations that are active in education or psychosocial support in the fields of palliative and end-of-life care. These collaborators were provided with the list of included publications, along with an electronic form on which they were invited to identify additional relevant resources on the topic of young people’s awareness of death and dying. One collaborator requested a phone meeting to share the findings of a report on death education in schools recently submitted to the Quebec Ministry of Health.^22^

This consultation process led to the identification of 12 additional publications, which were then assessed against the inclusion and exclusion criteria. Discussions with interested parties from the fields of education and bereavement support also enhanced the problem framing, particularly regarding local ADD practices and the discussion of findings.

## Results

In total, 35 publications were included in this scoping review: 23 empirical studies, 5 practice reports, 5 book chapters, and 1 doctoral dissertation. In the context of this review, empirical studies refer to publications reporting a data collection and analysis process (e.g. quantitative, qualitative, or mixed-methods designs), whereas practice reports refer to descriptive accounts or reflections by practitioners that present an initiative without a formal research design. These publications, dating from 2010 to 2023, originate from Europe (n=27), North America (n=6), and Asia (n=1); one additional publication is a Europe–North America collaboration. Nearly all selected publications (n=33/35) contributed to answering at least two research questions. Detailed information about each publication is provided in Table 4.

**Table 4.** Summary of the characteristics of the included writings and their contributions to research questions.

Reference	Country	Aims	Type (design if applicable				Discipline	Framework (if applicable	Population (sample size if identifiable	Research questions answered		
			Empirical	Practice story	Book section	Dissertation				Practices	Perspectives	Factors
Abras, 2013^23^	France	Reporting a “positive experience educating primary school children about death” (p.82)	​	x	​	​	Health sciences	n/a	Primary school students (23)	x	​	x
Argent, 2015^24^	UK	Presenting “one example of the way a child psychotherapy approach can be used in a school setting” (p.132)	​	x	​	​	Health sciences	CPOP^25^	Primary school students (24)	x	​	x
Bacqué, 2015^26^	France	How to listen, talk, and represent death and loss in school ?	​	​	x	​	Health sciences	n/a	Primary and High Schools	x	​	x
Beccaro, Gollo, Ceccon et al., 2014^27^	Italy	“Developing and piloting an in-depth intervention on severe illness and palliative care in high schools; assessing students’ interest in and knowledge of palliative care, and the overall impact of this experience; assessing the usefulness of intervention components and procedures in both teachers and students.” (p.2)	x (QN)	​	​	​	Health sciences	MRC^28^	High School students (89)	x	x	x
Beccaro, Gollo, Giordano, et al., 2014^29^	Italy	“Developing and piloting a school-based intervention on severe illness-induced bereavement through a project focused on spreading knowledge of palliative care among high school students” (p.756)	x (QN)	​	​	​	Health sciences	MRC^28^	High School students (159)	x	​	x
Berman, 2019^30^	USA	“Develop a model curriculum that would provide high school students with knowledge, understanding, and an appreciation of the Jewish preparation for burial; […] incorporate into the curriculum the core values of Judaism reflected in its death rituals through the use of sound educational principles; […] disseminate the curriculum” (p.6)	​	x	​	​	Education sciences	The Chevra Kadisha*	High School students (26)	x	​	x
Blake et al., 2020^31^	UK	“Evaluate the feasibility of implementing a public health intervention in schools to encourage pupils aged 12-15 years to independently explore ideas of death, dying, loss and end of life care in a structured and creative format.” (p.203)	x (IM)	​	​	​	Health sciences	PHPC^32^	High School students (24)	x	​	x
Case et al., 2020^33^	USA	“Exploring teachers’ experiences with discussing death in the classroom and educators’ feelings about their preparation to discuss death in the classroom.” (p. 399)	x (QL)	​	​	​	Apllied Communi-cation Studies	n/a	Teachers (19)	​	x	x
de la Herrán Gascón et al., 2021^34^	Spain	“Determine adolescents’ attitudes towards an education that takes death into account and includes it systematically into the curriculum.” (p.859)	x (QN)	​	​	​	Education sciences/Sociology/Social work	PoD^35^ DE^36^	High School students (1 897)	​	x	x
de la Herrán Gascón et al., 2022^37^	Spain	“Compiled the views of mothers and fathers, aiming to determine their attitudes to death education, the influence on these attitudes of a set of socio-demographic variables, and their opinions on how the topic of death is approached in society and schools.” (p.1321)	x (QN)	​	​	​	Education sciences/Sociology/Social work	PoD^35^ DE^36^	Parents (917)	​	x	x
Fawer-Caputo, 2015^38^	Switzer-land	“The subject of death appears very early [on in children’s questioning], and it is primarily the family, followed by teachers, who have the delicate task of answering them.” (free translation, p. 357)	​	​	x	​	Education sciences	n/a	Primary Schools	x	​	x
Friesen et al., 2020^39^	Canada	“Outlines the literature related to death education in the school, identifies and compares several death education resources for schools, and discusses the implementation of death education in the classroom. “ (p.332)	x (KS)	​	​	​	Health sciences	n/a	Schools’ curriculums (3)	x	​	x
Galende, 2015^40^	Spain	“Ascertain whether students from Teachers Training School have been or are being trained on Death Education; Test their knowledge and attitudes towards the inclusion of this subject in schools; Collect data about their religious beliefs, life experiences and attitudes towards death and Check whether that data relate to their attitude towards inclusion of the subject in schools” (p.93)	x (MM)	​	​	​	Education sciences	n/a	Teaching students (52)	​	x	x
Gatt, 2020^41^	Malta	“Investigate how children interpret the symbols of death present in the animated Disney movie ‘Coco’.” (p. 26)	​	​	​	x (QL)	Education sciences	SCP^42^	Primary school students (6)	x	x	x
King-McKenzie, 2011^43^	USA	“Explore two key issues: why death and dying in the curriculum, and how it should be integrated into the public-school curriculum.” (p. 512)	​	x	​	​	Education sciences	CuT^44^	Schools’ curriculums	x	​	x
Mak, 2011^45^	China	“Evaluate secondary students’ knowledge and attitudes about death and dying in Hong Kong” (p. 311)	x (QL)	​	​	​	Education sciences	n/a	High School students (30)	​	x	x
Markell, 2010^46^	USA	Exploring 4 topics: “(1) a brief history of the availability of death education for children; (2) objections to death education for children and counterarguments; (3) teacher and parent attitudes toward death and death education; and (4) death education in the schools.” (p. 293)	​	​	x	​	Education sciences	DE^47^	Primary school students	x	x	x
Martins Pereira et al., 2018^48^	Portugal	“Implement an education program about palliative care among teenagers and […] investigate the impact of the program on the participants.” (p.2)	x (MM)	​	​	​	Bioethic	HPPCA^49^	High School students (69)	x	​	x
Nikolakopoulou et al., 2014^50^	Greece	“Record elementary school children’ s level of knowledge, familiarity, and attitude towards the concept of death, in relation to their origin (urban or rural enviromnent); determine whether the above-mentioned parameters could alter after implementation of an appropriate intervention.” (p.110-111)	​	​	x (QN)	​	Psychology/Education sciences	n/a	Primary school students (191)	x	​	x
Orr and Henderson, 2020^51^	UK	“We enquired how we could best support and encourage young people to talk about death, dying and bereavement. We also wanted to know how we could help pupils to support each other when a family member, teacher or school friend are faced with a palliative illness.” (p.2)	​	x	​	​	Health sciences	APELCP^52^	High School students	x	​	x
Paul et al., 2016^53^	Scotland	“The research therefore aimed to explore the potential role of the Hospice in engaging with school communities” (p.2)	x (IM)	​	​	​	Social sciences/Health sciences/Education sciences	NPHAPC^32^	Primary school students/Parents/School staff (72)	​	x	x
Paul, 2016^54^	Scotland	“Explore and develop practice between a hospice and two primary schools.” (p.187)	x (IM)	​	​	​	Social sciences	NPHAPC^32^	Primary school students (34)	x	​	x
Paul, 2019^55^	Scotland	“Innovate the scholarly field of research into death and childhood studies while also provide a platform, underpinned by children’s perspectives, from which to inform death education and bereavement support.” (p. 557)	x (QL)	​	​	​	Social sciences	SA^56^	Primary school students (32)	​	x	x
Raccichini et al., 2023^57^	Italy	“The purpose of the death education workshop was, on the one hand, to address the themes of death and dying, familiarize participants with the topics of death and the end of life” (p.280)	x (QN)	​	​	​	Health sciences	n/a	High School Students (416)	x	​	x
Rodríguez Herrero et al., 2022^58^	Spain	“The present study addresses this gap in the existing research on death education: the investigation of attitudes and opinions toward death education among teachers at all levels of educational, using validated instruments and a broad sample.” (p.1519)	x (QN)	​	​	​	Education sciences/Psychology	DE^35^	Teachers (683)	​	x	x
Rodríguez Herrero et al., 2023^59^	Spain	“Explore how head teachers in preschool, elementary, and high schools understood education in the topic of death.” (p.1053)	x (QL)	​	​	​	Education sciences	DE^35^	Principals (7)	​	x	x
Scott et al., 2019^60^	Scotland and Canada	Reporting “on the findings from the study undertaken in Scotland which explored young people’s experiences of different types of loss, including the loss of someone close” (p.7)	x (MM)	​	​	​	Education sciences	n/a	High School Students (31) Parents (108) School staff (37)	​	x	​
Serrano Manzano et al., 2024^61^	Spain	“Determine perceptions of death education among parents of Spanish schoolchildren aged 3–18 year” (p.66)	x (QL)	​	​	​	Education sciences	PoD^62^	Parents (34)	​	x	x
Stylianou and Zembylas, 2018^63^	Cyprus	Two questions research: “(a) How do children perceive the concepts of grief and grieving; and (b) What was the effectiveness of an educational intervention that integrates those concepts critically and somewhat more systematically in the curriculum?” (p. 248)	x (IM)	​	​	​	Education sciences	HPSA^64^	Primary School Students (16)	x	​	x
Stylianou and Zembylas, 2021^65^	Cyprus	“Analyze teachers’ perceptions and affective experiences of an in-service training program on death education, which focused explicitly on teaching about the difficult issues of death, loss, and grief in the classroom.” (p.56)	x (IM)	​	​	​	Education sciences	DE^66^	Teachers (115)	​	x	x
Talwar, 2011^67^	Canada	“Address how adults in educational settings deal with such discussions with children, including the taboos associated with talking about death, educational professionals’ beliefs and attitudes toward discussing death with children, and the place of death education in the classroom and school.” (p. 98)	​	​	x	​	Education sciences	n/a	Teachers (59) Teaching students (60) Psychologist students (48) School psychologists (30)	​	x	x
Testoni, Biancalani et al., 2021^68^	Italy	“Verify whether the Italian project, entitled “Let’s Start With the End” (LSWE), was aimed to reduce, on the one hand, the denial or repression of someone’s death and, on the other hand, the anxiety deriving from mortality salience, improving the development of cognitive and emotional skills needed to deal appropriately with death-related thoughts.” (p. 732)	x (QN)	​	​	​	Health sciences/Social sciences/Psychology	TMT^69^; DE^70^	High School Students (227)	x	​	x
Testoni, Palazzo et al., 2020^71^	Italy	Explore “adolescents’ experiences of a death-education course by describing the effects in the first person” (p.2)	x (QL)	​	​	​	Health sciences/Social sciences/Psychology	TMT^69^; DE^70^	High School Students (215)	x	​	x
Testoni, Palazzo et al., 2021^72^	Italy	“Show that an experience of DE involving one hospice and its professionals may help students reduce alexithymia” (p.2)	x (MM)	​	​	​	Health sciences/Psychology	TMT^69^; DE^70^	High School Students (180)	x	​	x
Testoni, Ronconi et al., 2020^72^	Italy	“Confirm the efficacy of the distal defenses associated with TMT, namely how the representation of immortality could make the death anxiety elicited by the MS more manageable” (p.181)	x (QN)	​	​	​	Health sciences/Psychology/Art sciences	TMT^69^; DE^70^	High School Students (534)	x	​	x

***Notes***. APELCP: The Ambitions for Palliative and End-of-life care Partnership framework. CPOP: The Child Psychotherapy Outreach Project. CuT: Curriculum theory. DE: Death Education. Death education is not governed by a specific framework: many authors have contributed to its definition, but no consensus has been reached. Writings based on this theoretical framework therefore systematically refer to several authors or even propose their own definition. HPPCA: Health-promoting palliative care activities. HPSA: Holistic psycho-social approach. IM: Implementation study design. This research explores the implementation of interventions or educational approaches, most often through participatory methodologies. KS: Knowledge synthesis, systematized or not. MM: Mixed method study design. MRC: The Medical Research Council Framework for the Development and Evaluation of Complex Interventions n/a: non applicable. NPHAPC: New Public Health Approach to Palliative Care. PoD: Pedagogy of Death. QL: Qualitative design study design. QN: Quantitative design study design. SA: Sociological ambivalence. SCP: Sociocultural approach. TMT: Terror Management Theory

*A Chevra Kadisha is a Jewish “holy society” or burial society, a group of volunteers who prepare a deceased person’s body for burial in accordance with Jewish law and tradition.

**Figure 2.**
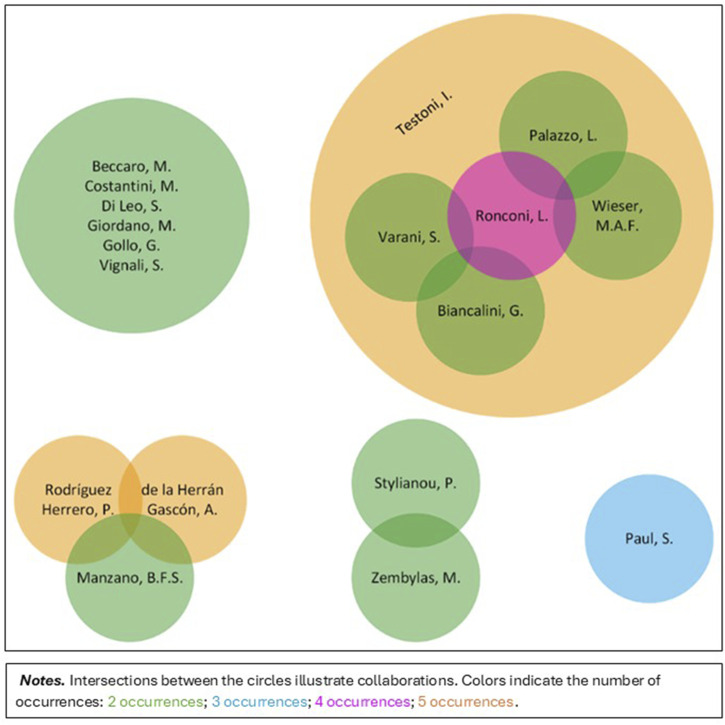
Co-occurrence network of authors appearing between two and five times in the corpus.

Figure 2 illustrates the recurrence of 18 authors who appear 2–5 times within the corpus. It shows that 17 publications involve at least one of these authors, highlighting regular collaborations. This concentration suggests that a relatively small number of research teams have been driving much of the work on ADD in school settings over the past decade.

## Q1. How do we raise awareness about death and dying in school settings?

Of the 35 publications reviewed, 22 of them have at least one awareness-raising practice addressing this first research question. Of these, 12 are empirical studies, including randomized or nonrandomized controlled trials (n=5/12),^57,68,71–73^ pilot interventions or implementation studies (n=3/12),^27,29,31^ action research (n=3/12),^48,54,63^ and knowledge synthesis (n=1/12).^39^ The remaining publications consist of practice reports (n=5/22),^23,24,30,43,51^ book chapters (n=4/22),^26,38,46,50^ and a doctoral dissertation^41^ reporting an action research project. Table 5 presents the awareness-raising practices identified in the literature. When available, details on practice duration and involved staff are also provided.

**Table 5.** Summary of awareness-raising practices reported in the included publications (type, duration, and practitioners involved).

​	Reference	School level	Intervention/Practice								Duration (period)	Practitioners involved in the practice
			Art	Lectures	Visit	Class discussion	Curriculum	Book	Film	Other (specify)		
Practice reports	Abras, 2013^23^	PS	x	​	​	x	​	​	​	​	6h (1 mo.)	Teachers
	Argent, 2015^24^	PS	​	​	​	x	​	​	​	​	1h (1 mo.)	Teacher Psychotherapist
	Berman, 2019^30^	HS	​	x	x	​	​	​	x	​	10h	Teachers Rabbis
	King-McKenzie, 2011^43^	n/a	​	​	​	​	x	x	​	​	u/s	Teachers
	Orr and Henderson, 2020^51^	HS	​	​	​	​	​	​	​	Information booth	u/s	Pastoral team Charity organization Specialized palliative care team
Dissertation	Gatt, 2020^41^	PS	​	​	​	x	​	​	x	​	∼ 2h	-
Book sections	Bacqué, 2015^26^	PS/HS	x	​	x	x	​	x	x	Breeding animals	u/s	Teachers Elderly people
	Fawer-Caputo, 2015^38^	PS	x	​	​	x	​	x	x	​	u/s	-
	Markell, 2010^46^	n/a	x	x	​	x	x	x	x	​	u/s	**-**
	Nikolakopoulou et al., 2014^50^	PS	x	​	​	x	​	​	​	​	u/s	**-**
Empirical studies	Beccaro, Gollo, Ceccon et al., 2014^27^	HS	x	x	​	x	​	​	x	​	u/s (4 mo.)	Teachers Palliative care nurses Psychologists
	Blake et al., 2020^31^	HS	​	​	​	​	​	​	​	Storytelling contest	u/s	Charity organization Community
	Beccaro, Gollo, Giordano, et al., 2014^29^	HS	​	x	​	x	​	​	x	​	∼ 4h	Teachers Bioethicist Palliative care nurses Psychologists
	Friesen et al., 2020^39^	n/a	​	​	​	​	x	​	​	​	u/s	Teachers
	Martins Pereira et al., 2018^48^	HS	​	x	​	​	​	​	​	​	3h	Reverend Community members Palliative care nurses
	Paul, 2016^54^	PS	​	x	​	​	​	​	​	Fundraising activities	u/s	Teachers Hospice
	Raccichini et al., 2023^57^	HS	x	x	​	x	​	​	​	​	8h(1 mo.)	Teachers Psychologist
	Stylianou and Zembylas, 2018^63^	PS	x	x	​	x	​	​	​	​	>10h	Teachers Priest
	Testoni, Biancalani et al., 2021^68^	HS	x	​	​	x	​	​	x	​	6h	Psychologist
	Testoni, Ronconi et al., 2020^73^	HS	x	x	​	x	​	​	​	​	5 to 10h	Psychologist Teachers
	Testoni, Palazzo et al., 2020^71^	HS	​	x	x	x	​	​	x	​	∼ 40h (5 mo.)	Psychologist Teachers Religious studies expert
	Testoni, Palazzo et al., 2021^72^	HS	x	x	x	​	​	x	x	Meeting with bereaved individuals and families of terminally ill patients	u/s (2 mo.)	Psychologists Monks Anthropologists Pediatric palliative care physicians

***Note.*** h: hours; HS: High school; mo.: months; n/a: non applicable; PS: Primary school; u/s: unspecified.

Among the cross-cutting themes identified across these publications, one concerns the role and influence of religion. On the one hand, this is reflected in the direct involvement of religious actors in the proposed practices (e.g., priests).^41,44,49,56,58,67,71,72^ On the other hand, religion (whether that of the young people, teachers, or parents) is sometimes used as a pre- or post-intervention variable,^62,70-73^ or even as a criterion for selecting participating schools,^60^ for instance to compare outcomes between religious and nonreligious schools. In several of these interventions, activities took place in denominational schools or in contexts where religious education is part of the curriculum, which may help explain the prominent role of religious actors and variables in these publications.

### Types of activities included in awareness-raising practices

The reviewed publications describe a variety of awareness-raising practices. Nearly half (n=16)^23,26,27,29,30,38,41,50,54,57,63,68,71–73^ combined at least two different activities, often designed to be complementary; for example, an art-based activity might be followed by a group discussion to reflect on the emotions that emerged.

Facilitating a discussion (e.g., philosophical questioning, debate) with students is the most frequently reported activity.^23,24,26,27,29,38,41,46,50,57,63,68,71,73^ In the writings that describe classroom discussions, these activities are reported to provide space for exploring various themes in accordance with students’ needs, such as previous experiences with death and illness, concerns about the body after death, and worries about parental mortality. Lectures also emerge as a recurring practice across the reviewed publications.^26,27,29,30,38,46,48,54,57,63,71–73^ These may take the form of didactic sessions led by professionals (e.g., nurses, psychologists, anthropologists), dialogues with bereaved individuals, or classes taught by religious representatives. Furthermore, art-based practices are particularly emphasized in the reviewed literature,^23,26,27,38,46,50,57,63,68,72,73^ including photovoice, role-play, and writing fictional letters. The use of media (films, documentaries, books) is also prominent,^26,27,29,30,38,41,43,46,68,71,72^ where these serve as pedagogical tools to launch students’ reflections on death and dying. Finally, some publications^26,30,71,72^ describe student visits to places of worship or hospices, and other publications^39,43,46^ present curricula or lesson plans designed to help teachers address death and dying with students according to their age and educational level.

### Practitioners involved

A wide variety of practitioners are involved in the practices identified, originating from both the school setting (e.g., teachers) and from palliative care (e.g., nurses, psychologists). Palliative care experts tend to be engaged primarily in “theoretical” activities, such as lectures and group discussions. Several publications also highlight collaboration with community organizations (e.g., retirement homes) or charitable organizations in the field of palliative care. For example, the practice report of Orr and Henderson^51^ describes how a high school partnered with a charitable organization and a palliative care team to develop activities on death and grief. Among the events organized, the authors specifically mention the facilitation of a booth where students were invited to talk to the palliative care team about three key questions addressing young people’s awareness of death and the support to be offered to a loved one at the end of life. Through this event, the palliative care team became integrated into the school community, which resulted in the subsequent organization of additional awareness activities and created opportunities for student volunteer involvement.

## Q2. What are the views of young people, parents, and school personnel on raising awareness about death and dying in school settings?

In total, 16 of 35 publications provide insights into this second research question. The majority are empirical studies (n=13/16),^27,33,34,37,40,45,53,55,58–61,65^ two are book chapters,^46,67^ and one is a doctoral dissertation.^41^ Of these, most (n=9/16) report on the perspectives of school personnel^27,33,40,46,53,58–60,65,67^ and/or young people (n=7/16).^27,34,41,45,46,55,60^ Only a few publications include parental views (n=4/16).^37,46,60,61^

### Young People’s perceptions of ADD

#### Death as omnipresent, yet unacknowledged

In conducting focus groups with young people aged 9 to 12 to explore their perceptions of death, Paul^55^ notes that they have had varied experiences relating to death. These experiences are omnipresent in some aspects of their personal, social, and educational lives, whether through literature they read at home or at school, or through documentaries, films, and video games they consume. Beccaro et al.^27^ reported that, prior to their educational intervention on severe illness and palliative care, 40.5% of high-school students stated that they had already heard of palliative care, based on an open-ended question about the WHO-defined approach. In this study, palliative care was used as the main entry point to talk about serious and life-threatening illness, end-of-life situations, and death, which overlaps with the broader issues addressed by ADD. Despite the frequency of these encounters with death, several writings report that the topic is rarely addressed openly with young people, except through initiatives led by certain actors.

This lack of opportunity for discussion is echoed in other studies,^41,45^ in which young people even describe death as a family taboo. According to Gatt^41^ young people’s curiosity drives them to seek answers from sources outside their family. Primary school students interviewed by Paul^55^ explicitly expressed a need for more open discussions about death in order to be better prepared for such life experiences. This desire to talk about death within the school context is also reported by Markell.^46^

#### Young People’s attitudes and preferences

In their study of 1,897 adolescents in Spain, de la Herrán Gascón et al.^34^ report a moderately positive attitude toward the integration of death into education. The authors note differences according to gender and age, with girls and older adolescents showing a more favorable attitude toward such an integration. Conversely, students from religiously affiliated schools and those identifying as religious were less supportive of raising ADD, preferring that this topic be addressed within religious institutions.^34^

In the writings included in this review, young people report that they prefer to discuss death primarily with family members, including parents, followed by friends.^34,45,60^ School staff, particularly guidance counselors and classroom teachers, are seen as secondary resources for such conversations.^34,60^ However, in the study by de la Herrán Gascón et al.,^34^ a majority of adolescents were either uncertain (40.3%) or did not believe (23%) that their teachers had received training on this subject.

When considering how to introduce death and dying in school, it is important to consider the cultural and social context in which young people live. In Mak’s qualitative study^45^ conducted with Chinese secondary school students, most participants expressed a desire to learn about death and dying to become more responsible, be better equipped to face life situations, and help prevent avoidable deaths (e.g., suicide). Most of them recommended that the topic be introduced in secondary school, when they feel more mature and able to address complex issues, with a higher level of abstraction. They reported wanting to discuss the meaning of life, death, and loss through group discussions or interactive activities embedded within existing subjects (e.g., science, humanities). Teachers, given the established relationship of trust, were perceived as legitimate facilitators for these activities, although young people also considered it relevant to involve other actors with personal or professional experience of the topic.

#### Adults’ perceptions

In the literature on parents’ and school staff’s perceptions, some similarities found help explain why death is rarely discussed with young people. Some parents report avoiding the topic to protect their children from distress,^60,67^ while others acknowledge struggling to manage their own emotions when confronted with it.^65,67^ Reflecting the proximity and continuity of education between home and school, several studies emphasize the need to prepare all adults – both school staff and parents – to engage with young people in discussions about death.^37,61^ Markell^46^ and Talwar^67^ further highlight the discomfort experienced by parents and teachers when addressing death and bereavement, as well as the challenges of explaining these issues to young people.

#### Parents’ perceptions

On the parents’ side, the overall attitude toward integrating ADD into school curricula is generally positive,^60,67^ under certain conditions. For instance, in some writings included, parents reported preferring that the topic be introduced with a secular approach, adapted to young people’s developmental stages.^37,61^ Some studies also stress the importance of ensuring that teachers are properly trained on these issues and of collaborating with psychological support or counseling services when these subjects are addressed.^46,61^ Parents in Manzano et al.^61^ study suggest incorporating ADD into existing curriculum subjects (e.g., literature, language, science) or as a cross-curricular theme within educational activities (e.g., film forums, field trips). On the other hand, some parents argue that death should primarily remain a family responsibility, particularly given the cultural aspect of the subject and the trust-based relationship between children and their parents.^46,60,61^

#### School staff perceptions

Across several studies conducted in European and North American contexts,^33,40,46,58–60,65,67^ school staff (teachers, pedagogical counselors, school psychologists, and principals) tend to express moderately positive attitude toward ADD, while also highlighting important reservations and barriers. They emphasize the importance of opening up the discussion on topics that are now largely absent from curricula because of their taboo status, while also stressing the need for secular and evidence-based approaches.^58,67^ Some of the barriers reported in the writings include a lack of emotional and pedagogical preparation, along with concerns about parental reactions,^65^ with some teachers considering death education to be primarily a family responsibility.^40^ Despite these reservations, some teachers feel competent to guide students’ reflections on death,^58,59^ although they also acknowledge having insufficient training.^33,40,46,60,65,67^ This paradox, particularly evident when addressing death explicitly in the classroom, leads Rodríguez Herrero et al.^58^ to question the reliability of teachers’ self-assessments of their competencies in ADD.

## Q3. What factors influence such awareness-raising in school settings?

**Figure 3.**
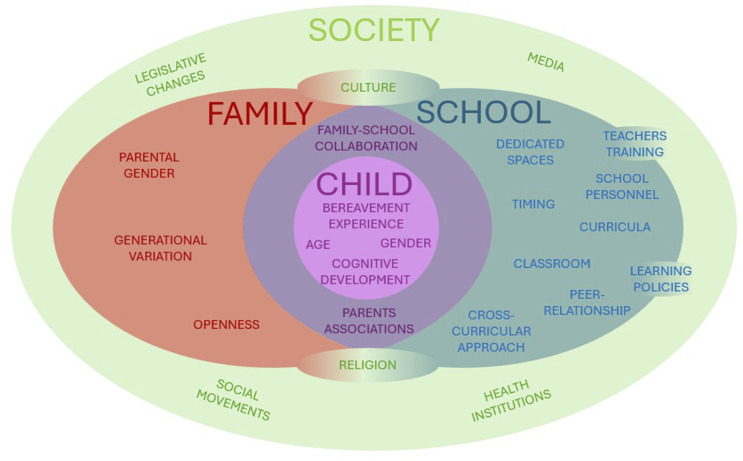
Illustration of factors influencing awareness of dying and death in school settings.

Almost all the publications retrieved (34/35) mention numerous factors influencing ADD in school settings. These factors influence various actors’ perceptions of ADD or practices in development. To better characterize the various factors, we categorized them by the environments with which young people interact: family, school, and society. Figure 3 offers an illustration of these factors and their relationships.

### Individual characteristics

Many writings identify individual characteristics in young people that play a role in their perception of ADD, namely, their age, cognitive and socioemotional development, gender, and bereavement experience. As mentioned previously, across several writings older children tend to be more supportive of integrating ADD into schools,^34,40,41,43,68^ though they are less likely to recognize its educational value.^34^ In the reviewed writings, the understanding of death as being both irreversible and universal develops from early childhood, but fully grasping its implications is portrayed as requiring a certain level of cognitive maturity.^24,26,38,40,41,43^ In some studies, girls are reported as being more favorable to ADD than boys,^34^ while also expressing greater fear of death.^68^ Finally, personal bereavement experiences (e.g., attending funerals, loss of a pet) also shape attitudes toward and understanding of death and dying, as documented in several writings.^26,34,45,57,58,63,67,68^

#### Family environment

Within families, some studies report parents as being open to discussing dying and death,^37^ although children sometimes report that such discussions are avoided or minimized by their parents.^41^ These findings come mainly from studies conducted in European and North American contexts, where family roles and expectations regarding discussions about death may differ from other cultural settings. On a relational level, some young people are often hesitant to talk about death for fear of upsetting or worrying those around them, which restricts discussion and limits their freedom of expression.^43,55^ The study of de la Herrán Gascón et al.^37^ showed that parent gender also influences openness to including ADD in school curricula, with mothers generally showing more willingness than fathers to do so. Gatt^41^ additionally notes a generational variation: grandmothers are perceived as less willing than parents to discuss death and dying.

Mutual trust and collaboration between parents and schools are described as essential components in awareness-raising practices.^65,67^ Due to the perceived *sensitive* nature of the topic and the administrative and ethical procedures specific to each school, most studies highlight the need to obtain explicit parental consent before any activity related to death and dying.^24,39,41,46,54,63^ The leadership of parent associations can also facilitate family engagement and strengthen the climate of trust surrounding conversations on death and dying.^61^

#### School environment

School is an environment where young people spend much of their time, socialize, and learn.^26^ This environment influences ADD both through the establishment’s physical structure and curriculum, and through the people who interact with it, including students (peers) and school personnel.

In some practice reports, it is deemed important to have dedicated spaces^23^ for expressing emotions and representations related to death, that are strategically positioned and accessible (e.g., frequently used areas).^51^ In the pilot study of Beccaro et al.,^27^ classrooms are perceived by young people as safe spaces, conducive to opening up discussion about death and dying. Beyond the school’s physical environment, learning policies and curricula are repeatedly identified as major factors influencing the integration of ADD into the school environment.^39,45,50,54,67^ Friesen et al.^39^ highlight that variations in policies and curricula across schools in Canada result in unequal implementation of activities. Paul et al.^53^ add that without political intention to formally integrate ADD into educational curricula, initiatives remain fragmented. Several writings^26,38,39,45,50,54,67^ therefore recommend that death and dying be included as topics in curricula to 1) ensure consistency in the messages conveyed to young people, 2) facilitate parental consent and engagement, and 3) ensure the sustainability of initiatives. A cross-curricular approach is often suggested, by integrating death and dying into existing subjects (e.g., history, art, music, biology).^26,38,45,50,61,67^ Timing is also important: activities scheduled at the end of the day may increase anxiety and reduce discussion quality,^24^ whereas activities held after exam periods are over support better emotional and organizational availability.^30,51,68^

In terms of the people included in the school environment, peer relationships are described as providing space for expressing emotions, identifying experiences, mutual understanding, and comparisons,^23,27,38,51^ which, according to Paul^55^ can counterbalance the protective dynamics established by adults. This space is shaped by several factors: the rarity and sensitivity of spontaneous discussions,^43,71^ a climate of trust among children,^65^ and the dynamics of sharing and openness.^72^ School personnel, including teachers, psychologists, and educators, play a central role in ADD due to their daily contact with students.^24,26,27,29–31,38,40,41,48,54,57–59,65,67,72^ In many writings, student engagement with ADD is facilitated by teachers’ professional stance, namely, an open, caring, and professional attitude,^27,29,72^ combined with participatory and creative activities.^26,30,31,48,65^ Teacher involvement is sometimes reported as limited by lack of time or motivation,^29,54,57^ or by difficulty in managing their own emotions and dilemmas relating to death and dying.^40,65,67^ Finally, certain sociodemographic characteristics of school personnel (e.g., gender, age, years of experience, teaching level) also appear to influence their attitude toward ADD in schools.^58^

Teacher training emerges as a factor influencing perceptions and practices on ADD. Several writings^26,33,40,45,48,59,63,65,67^ note the lack of specific training on death, despite recognition of its educational relevance. In the reviewed literature, teachers express the need for initial, regular, and ongoing training on death and dying, children’s and teenagers’ emotional health, and how to address the topics with students.^33,43,45,48,59,63,65,67^

### Cultural and social environment

On a broader scale, some characteristics of the society in which young people evolve influence ADD, notably, the religious and cultural context and the way in which death and dying are talked about in social discourse. A recurring factor mentioned in the writings is the religious context.^24,34,37,38,41,45,53,58,68^ Young people’s personal beliefs, often linked to their spirituality, influence their responses to ADD practices.^68,73^ For instance, secondary school students acknowledge that their own beliefs and those of their parents shape their understanding of death.^45^ Several empirical studies indicate that atheist parents, children, and teachers are more favorable to integrating death into school education than their Catholic counterparts.^58,61,67^ Conversely, Talwar^67^ observes that highly religious individuals feel more comfortable addressing the topic. The school’s religious affiliation is also mentioned in multiple writings, although findings are sometimes contradictory: students in nondenominational schools tend to respond more positively,^34^ whereas teachers in denominational schools report more favorable attitudes toward its curricular integration.^58^

Culture also shapes how ADD is perceived.^45,61,72^ For example, migration experiences may destabilize young people’s social references, making existential discussions more challenging in an unfamiliar sociocultural environment.^24^ Cultural differences also emerge in the use of terminology and symbolic references. For example, in some Chinese communities, the term “life education” is preferred over “death education” to make such practice more culturally acceptable.^45^ Moreover, Gatt^41^ study indicates that children in Malta might struggle to interpret Mexican cultural representations like those illustrated in the film *Coco.*

News, entertainment, and social media shape young people’s perceptions of death and dying.^26,38,45,72^ Some parents express concern about violence in some video games and the idealized lifestyles promoted on social media, both of which may influence how young people understand death and dying.^26,38,61^ They also note that media and health institutions contribute to a hegemonic view of death, where it is either hidden or perceived as a medical failure rather than a natural life stage.^61^ Certain social movements can mark a turning point in the place given to death and dying in society,^46,48^ influencing educational practices. For example, the *standards movement* may limit the space available to introduce death and dying practices, by reducing curricula to basic competencies,^46^ whereas major legislative changes, such as the legalization of euthanasia in Portugal, may have created a context favorable to broader discussions on the topic.^48^

## Discussion

This scoping review explored how ADD is raised in school settings, how it is perceived by young people, parents, and school staff, and which factors influence such practices. Our synthesis shows that schools host a wide range of multimodal practices to address ADD, that attitudes toward these initiatives are mostly positive across those groups, and that ADD in schools is deeply shaped by individual, familial, institutional, and sociocultural factors.

Various awareness-raising practices combining multiple activities were identified in the writings, emphasizing the value of multimodal approaches that engage cognitive, emotional, and creative dimensions. The literature also shows the importance of interdisciplinary collaboration and community engagement in implementing such practices in school settings. Although our results regarding ADD perceptions reveal generally positive attitudes, there are nuances among the three groups of actors (young people, parents, and school staff). While this remains a minority view, it is important to note that some participants (young people and adults) believe that death and dying should be addressed primarily within the family unit. Many of the writings included in this review stress the importance of approaching death and dying in schools from a secular perspective that is sensitive to young people’s diverse beliefs, while also adapting content to their cognitive and emotional development. While school is identified as a relevant and secure environment for opening discussions on death and dying, it appears necessary for teachers to be trained to adequately support students. Since our database search was completed (June 2023), a scoping review by Riera-Negre et al.^74^ has further synthesized support strategies and training needs for teachers facing illness, bereavement, and death-related challenges in the classroom. While their focus is more specifically on teachers’ support strategies than on young people’s awareness of death and dying, their findings converge with ours in underlining the central role of structured training, institutional guidance, and context-sensitive approaches. However, while our results suggest that school personnel are interested in integrating ADD into their curriculum, provided they receive training, some contextual factors may lead to different role expectations. For instance, a report^22^ shared by one of our collaborators recommends that grief specialists, rather than teachers, be primarily responsible for addressing the subject of death and dying with young people.

Our results identify numerous factors that influence ADD, showing the multidimensionality and systemic nature of this phenomenon. Talking about death and dying with young people is invariably part of the social and cultural norm system surrounding a given child, their family, and the school system. One of the factors that illustrates this systemic nature is religious affiliation. Religion is a familial and societal factor that influences how people perceive and react to death and dying, as individuals but also as members of the school community. Finally, our analysis of the factors reminds us that ADD is not solely a classroom or family issue: without clear political and institutional support, such practices remain fragile and dependent on isolated actors.

## The *taboo* of death: Myth, narrative, and the role of schools

Like scientific and social discourse, the identified literature frequently refers to death as a *taboo*, an idea that stems from the theory of death denial^75^ and refers to an implicit prohibition on certain behaviors and practices.^76^ However, recent studies have questioned this theory, clarifying that individuals are willing to talk about their own mortality but fear the reaction of their loved ones.^77–80^ The *taboo* is more a social narrative used to explain reluctance than a universal reality.^81–83^ This taboo is reflected in our findings, with all interested parties saying they are in favor of integrating ADD into schools, but at the same time fearing that it will make others feel vulnerable, which helps maintain the status quo. These differences in perception reflect a structural barrier and serve as a reminder that ADD must be part of a relational dynamic and a dialogue between young people, parents, school staff, and the community.

As in sexual health education,^26,38,84^ the writings we reviewed also report resistance related to the perceived “sensitivity” of the subject, the requirement for parental consent, or the preference for dealing with it within the family sphere.^26,37–39,46,60,61^ However, schools can become a structured space for mediation on *sensitive* or *taboo* subjects, if teaching methods are adapted, teachers are trained, and education policies provide a clear framework^26,38,84–87^ – conditions that are also highlighted by our analysis.

## Between academic subjects and life lessons

The results of this scoping review also suggest that ADD can be integrated into existing courses.^26,38,39,45,50,54,67^ This approach is consistent with cross-curricular skills,^88^ which aim to overcome the compartmentalization of school subjects in favor of holistic and interdisciplinary teaching that promotes critical thinking, autonomy, and problem-solving in everyday life.^88–93^ It also calls for adapting interventions to students’ cognitive development stage,^88^ a recommendation of the current scoping review and another recent publication.^22^ Beyond the transmission of knowledge, the curricular integration of ADD could become a lever for young people’s overall development and their preparation for life in society.

Our findings also show that ADD is deeply linked to its place in cultural and social structures. While secularization predominates, the resurgence of fundamentalist movements – a minority but a visible one^94^ – can hinder the discussion of sensitive topics in schools (e.g., death, gender equality, individual rights) and reinforce discriminatory norms.^95,96^ In such situations, teachers fear contradicting family beliefs,^54^ thereby undermining the school-family collaboration needed to integrate such subjects. ADD can therefore only be effectively implemented if the parties involved engage in a joint dialogue on their collective, evolving, and contextual values.

Thus, ADD lies at the intersection of two complementary perspectives. On the one hand, it can be viewed as a specific subject requiring specific declarative knowledge. On the other hand, it can be integrated as cross-curricular learning, contributing to the development of existential and civic skills, and from this perspective, to death literacy itself.^16^ Indeed, several findings from this review align with the core components of death literacy as described by Noonan et al.^16^: the value of multimodal and participatory approaches, the emphasis on safe spaces for dialogue, and the importance of community engagement all reflect the knowledge, skills, and community resources that enable individuals to act in death-related situations. While the studies included in this review rarely mobilized the death literacy framework explicitly, their outcomes can be read through this lens, suggesting that ADD practices in schools may constitute an early and promising pathway toward death literacy in younger populations. Schools would then become a privileged place for familiarizing young people with the language, symbols, and spaces for dialogue around the finite nature of existence, strengthening their autonomy, resilience, and ability to engage in a more open and supportive conversations about death and dying within their families, schools, and communities.

## Strengths, limitations, and future research

This scoping review is, to our knowledge, the first to systematically map, within a single synthesis, school-based practices, the perspectives of young people, parents, and school staff, and the multi-level factors that influence ADD in school settings. In doing so, it moves beyond isolated reports of death education initiatives to offer an integrated, system-level view of how ADD is currently framed and enacted, and how it can be read through the lens of death literacy. The findings can guide school staff in implementing ADD, and they highlight the responsibility of policymakers. The systematic methodology, the team’s interdisciplinary expertise, and its collaboration with community partners all strengthen the rigor and relevance of the results. Moreover, by situating these findings within broader societal debates, this review contributes to ongoing discussions about a socially significant issue.

This review also has limitations. Grey literature is likely underrepresented, since everyday pedagogical practices are often difficult to access or poorly documented. Future research with a broader scope, conducted directly with teachers and schools (e.g., surveys) could provide a more comprehensive mapping of practices, thereby reinforcing collaboration between researchers and educational communities and ensuring continuity between theory and practice. Despite a rigorously validated search strategy, developed in collaboration with a disciplinary librarian, the available literature reflects important gaps. The corpus is predominantly Western and high-income: only one non-Western study, from China, was included. Similarly, the religious contexts represented are predominantly Christian (most often Catholic) with some references to Jewish educational settings; perspectives from other faith traditions are largely absent. Taken together, these cultural, geographical, and religious constraints mean that our findings reflect a specific landscape, and that their transferability to other contexts calls for caution. A further limitation is that a relatively small number of research teams account for a large share of the publications in this field. This concentration may contribute to the relative homogeneity of certain approaches and underscores the importance of supporting more diverse research initiatives on ADD in schools, in terms of teams, settings, and theoretical frameworks. Finally, this review reflects the state of knowledge up to June 25, 2023; more recent initiatives or publications on ADD in schools fall outside the temporal scope of our search and were therefore not included.

In conducting this research, we found that studies explicitly linking ADD to death literacy remain rare. While some writings used death education or death pedagogy frameworks, reflecting an interest in developing knowledge related to death, the fact remains that accumulating knowledge is not enough to enable involvement in death and dying situations. One explanation may be that many publications included in this review predate the formalization of the death literacy concept, which may explain why authors did not explicitly frame their findings in these terms. Nonetheless, several describe outcomes – such as increased knowledge, communication skills, and engagement with community resources – that can be interpreted as components of death literacy, even if they were not framed in these terms. Future research should explicitly mobilize the death literacy framework to design, implement, and evaluate ADD practices in school settings.

## Conclusion

This scoping review underscores that ADD in school contexts is embedded within a complex network of interrelated influences across individual, family, school, and broader sociocultural levels. In this perspective, active collaboration among all parties involved therefore constitutes a key lever for the implementation of sustainable ADD initiatives in schools. While ADD can be integrated into existing curricula in line with cross-curricular competencies, its success necessarily depends on the continuing education of school staff, the contextual adaptation of activities, and the establishment of a clear policy framework. Finally, this review points to several avenues for future research, likely to test and refine these propositions in diverse cultural and educational settings, and to make explicit how different methodological approaches shape what we can know about ADD in schools. Because death is an inevitable reality, integration of this subject in schools can provide young people with the tools to think and talk about death, thereby preparing tomorrow’s citizens to face life’s realities with autonomy, resilience, and openness.

## Supplemental material

Supplemental material - Youth awareness on death and dying in school settings: A scoping review on knowledge and practices Supplemental material for Youth awareness on death and dying in school settings: A scoping review on knowledge and practices by Emilie Allard, RN, PhD, Clémence Coupat, RN, MSc, PhD, Dimitri Létourneau, RN. Ph.D, Gabrielle Fortin, TS., PhD, Olivia Nguyen, MD, MM, Sabrina Lessard, Ph.D in Palliative Care and Social Practice

## Data Availability

Data can be available upon reasonable request to the first author of the study.*
